# Novel sustainable filter for virus filtration and inactivation

**DOI:** 10.1038/s41598-022-13316-9

**Published:** 2022-06-01

**Authors:** Tanya Purwar, Helber Antonio Esquivel-Puentes, Venkatesh Pulletikurthi, Xing Li, Ali Doosttalab, Clarice E. Nelson, Rita E. Appiah, Ernest R. Blatchley, Victor Castano, Luciano Castillo

**Affiliations:** 1 School of Mechanical Engineering, Purdue University, West Lafayette, IN 47906 USA; 2 Department of Agricultural and Biological Engineering, Purdue University, West Lafayette, IN 47906 USA; 3 Lyles School of Civil Engineering, Purdue University, West Lafayette, IN 47906 USA; 4 Division of Environmental and Ecological Engineering, Purdue University, West Lafayette, IN 47906 USA; 5 Centro de Fisica Aplicada y Tecnologia Avanzada, Universidad Nacional Autonoma de Mexico Boulevard Juriquilla, 76230 Queretaro, Mexico

**Keywords:** Mechanical engineering, Fluid dynamics, Viral infection, Disease prevention

## Abstract

The COVID-19 pandemic has caused a multi-scale impact on the world population that started from a nano-scale respiratory virus and led to the shutdown of macro-scale economies. Direct transmission of SARS-CoV-2 (Severe Acute Respiratory Syndrome Coronavirus 2) and its variants through aerosolized droplets is a major contributor towards increasing cases of this infection. To curb the spread, one of the best engineered solutions is the use of face masks to prevent the passage of infectious saliva micro-droplets from an infected person to a healthy person. The commercially available masks are single use, passive face-piece filters. These become difficult to breathe in during strenuous activities. Also, they need to be disposed regularly due to accumulation of unwanted particulate and pathogens over time. Frequent disposal of these masks is unsustainable for the environment. In this study, we have proposed a novel design for a filter for enhanced virus filtration, better breathability, and virus inactivation over time. The filter is called Hy–Cu named after its (Hy) drophobic properties and another significant layer comprises of copper (Cu). The breathability (pressure drop across filter) of Hy–Cu is tested and compared with widely used surgical masks and KN95 masks, both experimentally and numerically. The results show that the Hy–Cu filter offers at least 10% less air resistance as compared to commercially available masks. The experimental results on virus filtration and inactivation tests using MS2 bacteriophage (a similar protein structure as SARS-CoV-2) show that the novel filter has 90% filtering efficiency and 99% virus inactivation over a period of 2 h. This makes the Hy–Cu filter reusable and a judicious substitute to the single use masks.

## Introduction

For centuries, respiratory viruses have been a common cause for initiating epidemics and pandemics worldwide^1^, and the wearing of masks has a long history associated with preventing wide spread of such contagious pathogens. For example, in the 1918 Influenza epidemic, mandatory masking orders were passed around the globe^2^. In the 1980s and 1990s, with the outbreak of Avian influenza, wearing masks was taken as a preventive measure to curb the spread of respiratory infections. When the H1N1 flu hit Japan in 2009, wearing masks became a mandatory lifestyle choice and was adapted to normal living for a long time. In 2019, the first case of COVID infection was reported in China due to a respiratory virus called SARS-CoV-2^3^. Research shows that the infection occurs due to airborne transmission, when an infected individual exhales the virus into air while talking, coughing, or sneezing. The viruses also get suspended on common inanimate surfaces. The discharged virus can enter into a healthy individual’s respiratory tract through inhalation of the infectious micro-droplets in air or through hand-to-nose transmission after touching the contaminated surface. Several evidence based studies were conducted to determine the effectiveness of mask mandates^4^, and positive results were obtained^5^^,^^6^ showing that mask use is one of the best potential solutions to curb the virus spread. The available mask options to general public are N95 and KN95 masks, surgical masks, and cloth masks^7^. Although a cloth mask prevents the spread of large saliva droplets in the air^8^, its efficiency in preventing virus is still questionable^9^. The surgical masks are loose fitting, disposable devices that consist of two sheets of non-woven layer with a meltblown polypropylene layer in the middle, that is responsible for most filtration^7^^,^^10^. The N95 and KN95 respirators are designed to achieve a very close facial fit and form a seal around the nose and mouth. The main filtration material on these masks is an electrostatic non-woven polypropylene fiber^7^. The filtration efficiency of the KN95 is reported to be close to the medical grade N95 mask, i.e., up to 95% for 0.3 µm particles and larger, with a mean efficiency of 98%. Previous studies^11^ have shown that N95 masks have a filter resistance (*R*_*f*_) of 343 Pa for inhalation and 245 Pa for exhalation, respectively. Surgical masks are widely used due to their low air resistance because of their lower number of layers and thickness as compared to KN95. Their *R*_*f*_ and collecting efficiency have been characterized as 29 Pa and 32.9% respectively^12^.

Both KN95 and surgical masks are efficient in virus filtration, however, they need to be disposed because of their short usable life. This causes generation of large amounts of PPE (Personal Protective Equipment) waste^13,14^ that is also a potential source of micro-plastic pollution^15^. Another concern is the need for safe disposal of single use masks^16^ due to stable persistence of virus on these masks for days, that can cause unaccounted cases of virus infection if not handled and discarded properly. Studies have shown that when these pathogens are filtered and trapped in mask fibers, they can survive in between the layers for days which makes the masks hazardous for disposal with the non-hazardous waste^4^. The above limitations with commercially available and widely used engineered masks, form the motivational ground for our current study where the purpose is to design and test a novel sustainable filter that can achieve high breathability, longer life with re-usability, and better filtration efficiency.

The novel Hy–Cu filter proposed in this study composes of three unique layers that are tested in different configurations. The first unique layer is a spun-bond polypropylene material, coated with nano-engineered hydrophobic and lipophobic coating which is a water-based omni-phobic solution^17^. The coating, due to its hydrophobic properties, rejects the aerosolized micro- droplets of saliva and mucous, a common carrier of the virus^18^. The lipophobic property of the coating helps in preventing the virus from hosting on the fibers of the external layer, since the outer envelope of the virus structure, like most respiratory viruses is lipidic in nature. This outer lipid envelope holds the RNA together^19^ and is also susceptible to mechanical and environmental stress. The rupture of this protective lipid layer exposes the vulnerable RNA of the virus^20^ and causes inactivation. Another significant layer is a DLC (Diamond-Like Carbon) coated copper mesh. Copper is well known for its anti-microbial/anti-viral properties and capability to attack and deactivate pathogens^21,22^. Borkow et al.^23^ shows that if respiratory protective face masks are impregnated with copper oxide, it imparts in them anti-influenza biocidal properties without altering their physical barrier properties. The deactivation mechanism works when the pathogen comes in contact with the copper surface. The metal surface releases ions that alter the morphological structure of the pathogen. Moreover, these ions can break through the membrane of the bacteria or virus leading to inactivation and decontamination over time^24^. Buchegger et al.^25^ found that the DLC coating doped with zinc oxide (ZnO) is a promising material that could be used for pathogen rejection and deactivation^26^. The properties of the DLC are its low surface roughness, very high resistance to wear and tear, pH sensitivity, hydrophobicity, and super-hydrophobicity in some cases^27^. In a particular case when the DLC is doped with ZnO particles, zinc ions are released causing important anti-microbial actions in infectious environments with a pH in the range between 7.4 and 5.4.

In this study, performance of the proposed filter is analyzed in terms of a pressure resistance test, a virus filtration test and a virus inactivation, as well as a fluid resistance test, using well controlled experimental techniques. The experimental setup and methodology for finding *R*_*filter*_ and virus log reduction value (LRV) for the novel filter is discussed in the “Experimental Study” Section. The results on experimental and computational observations are discussed in “Results and Discussion” Section. The conclusions are given in “Conclusion” Section. The numerical setup for pressure resistance is discussed in Supplementary Note 1 and the fluid resistance test is discussed in Supplementary Note 2 in the Supplementary Information.

## Materials and method

### Filter fabrication and design

**Figure 1:**
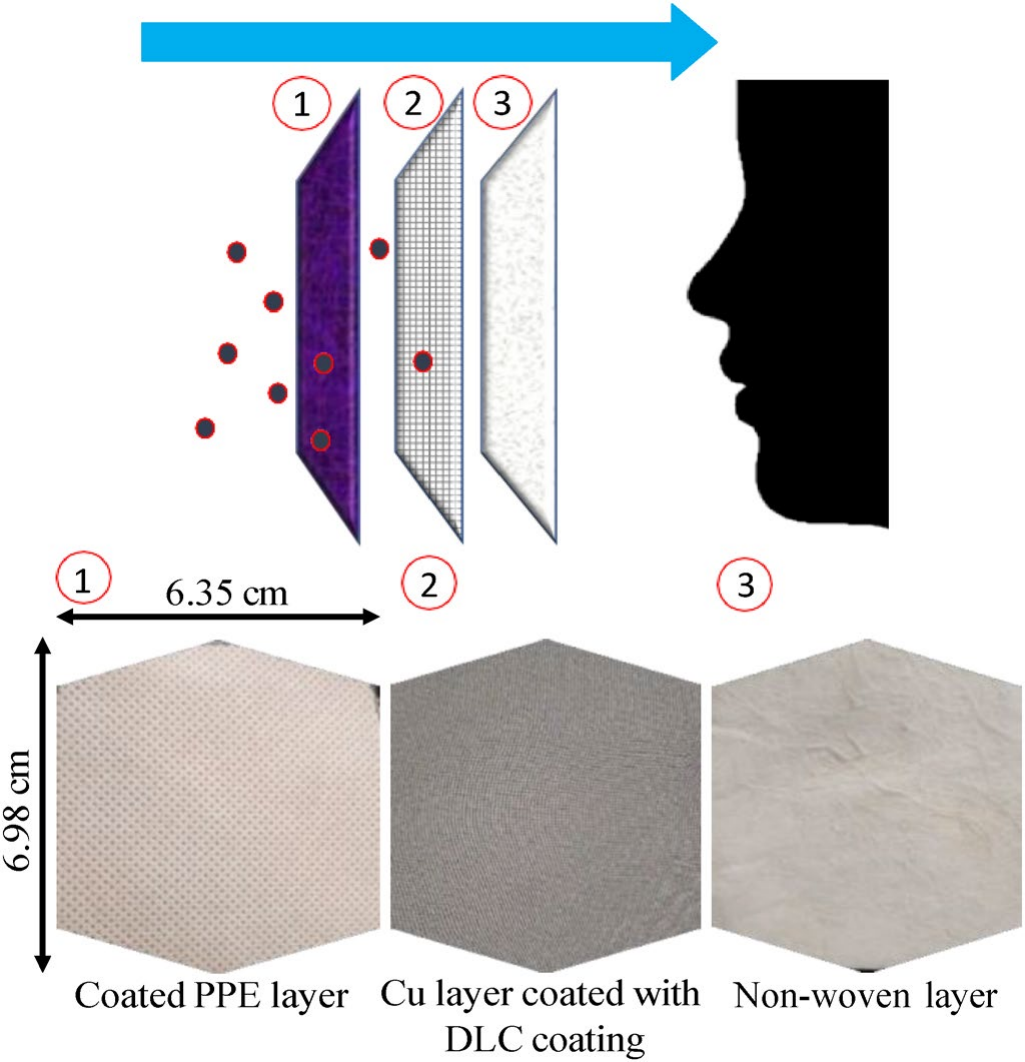
Layers of the proposed Hy–Cu filter.

In this study, the proposed novel filter has three unique layers and has been tested for efficiency in multiple configurations. Figure 1 shows the three unique layers, namely, omni-phobic coated polypropylene (PPE) layer, copper coated DLC layer and non-woven layer. The spun-bond polypropylene layer is coated with nano-engineered omniphobic water-based solution that is hydrophobic and lipophobic in nature. The layer is coated under controlled conditions using electrostatic spray deposition technique, ensuring uniform layer of hydrophobic and lipophobic coating. This increases the surface wettability of the outermost layer in the novel filter. The measurement of contact angle of artificial saliva droplet on the outermost layer of the Hy–Cu filter, surgical mask and KN95 mask is shown in the Supplementary Fig. S1. It is observed that the outermost layer of the Hy–Cu filter has comparable hydrophobic character as the KN95 mask and is better than a surgical mask. As previously discussed, the airborne transmission of coronavirus depends on the respiratory virus particles hosting on saliva or mucous aerosols/micro-droplets. The major function of the omniphobic coated layer is to repel these micro droplets. In case the virus particle reaches the surface of the exposed external layer, the lipophobic nature of the coating will prevent the virus from further entering the system. In terms of safety of use, the main components of the omniphobic coating are siloxanes and derivatives, (http://nctmexico.com.mx/.) as described in the corresponding patent and data sheets^17^. Generally, siloxanes are well tolerated by the humans, and they are an integral part of innovative methods of health care and nursing. They are commonly considered as minimal to non-toxic to humans and environment^28^. Data is primarily found on the cyclic siloxanes D4 and D5 and the small linear HMDS (hexamethyldisilazane). Siloxanes have a relatively low order of acute toxicity by oral, dermal and inhalatory routes and do not require classification for this effect. They are not shown to be irritating to skin or eyes and are also not found sensitizing by skin contact. Data on respiratory sensitization have not been identified^29^. The middle layer, diamond-like carbon (DLC) coated copper mesh has a pore size of about 200 micrometers. The coating was prepared by Technometals in Dayton, OH. Hydrogenated carbon and diamond-like carbon can be obtained using multiple techniques like chemical vapor deposition (CVD) or physical vapor deposition (PVD)^30^. For this study, the PVD technique was used^31^. The DLC coating on the copper mesh has a thickness in the range of 1–5 µm. The DLC coating is well known as bio-compatible^32^ material used in implants^33^, biological applications^34^, dental drills^35^, heart valves^36^, and many other applications directly used in humans^37^. The innermost non-woven layer, close to mouth is made of light polypropylene fabric, and has good air permeability, is breathable, non-toxic, and non-irritating. This non-woven interfacing is characterized based on mass per unit area. These textiles have fibers that are held together by hydro-entanglement with random mechanical intertwining of the filaments. In a non-woven fiber, the particulate removal takes place according to different mechanisms depending on particle size, namely, straining or sieving, inertial impaction, interception, and Brownian diffusion^4^. Different grades of non-woven layers affect the filtering efficiency. With higher mass per unit area, better filtration is expected.

Two different configurations of the Hy–Cu filter were prepared to test for pressure resistance efficiency, as well as virus filtration efficiency. For former experiments, a 3 layer (Hy–Cu 3 layer) and 5 layer (Hy–Cu 5 layer) configuration was tested. In the 3 layer arrangement, there is an omniphobic layer (outermost, exposed to atmosphere), followed by a DLC coated copper layer (middle), and a non-woven layer (innermost, close to mouth). In the 5 layer configuration, there is an omniphobic layer (outermost), a non-woven layer, followed by a DLC coated copper layer (middle), and 2 non-woven layers, thereafter, closest to the mouth. In the 5 layer configuration, the purpose is to sandwich the middle DLC layer between two non-woven layers to expose viruses trapped in non-woven layers to the anti-microbial DLC layer for maximum inactivation of the viruses. For virus filtration test, we studied the 5 layer configuration of the Hy–Cu filter.

### Experimental study

Three standard tests are used to determine mask effectiveness to curb the spread of respiratory viruses. The standard tests that have been used to study the efficiency of the novel Hy–Cu filter include a pressure resistance test, a virus filtration test and a virus inactivation test. A qualitative test to understand the fluid resistance is also conducted and discussed in the Supplementary Note 2 in Supplementary Information.

#### Pressure resistance test

**Figure 2:**
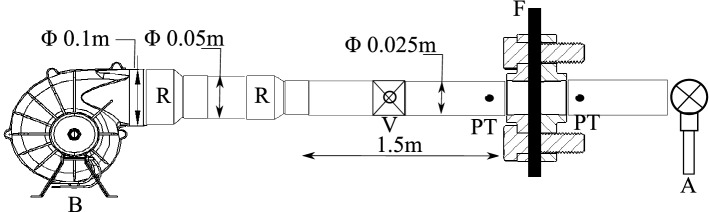
Schematic diagram of the pressure resistance test setup (B: Blower^39^, R: Reducer, V: Valve, PT: Pressure Taps, F: Filter/Mask, A: Anemometer).

Figure 2 shows the experimental setup that is designed to measure the pressure resistance offered by a mask or a filter at wide range of flow velocities such as, 0*.*5 m/s, 1*.*9 m/s, 3*.*5 m/s, 7*.*5 m/s, 9 m/s, 11 m/s, 12*.*5 m/s, 14*.*5 m/s. These velocity cases are decided based on human respiratory actions^38^ of breathing at about 1 m/s, talking at 5 m/s, coughing at 10 m/s and sneezing at 20 m/s. The filter/ mask samples that were tested for pressure resistance include a KN95 mask, a surgical mask, the Hy–Cu 3 layer filter and the Hy–Cu 5 layer filter. A 12V DC voltage blower with maximum flow rate of 178 m^3^/h was used to create air flow through the filter. Reducers were used to decrease the blower outlet diameter from 0*.*1 to 0*.*025 m. A ball valve was used to control the flow rate for various air speeds and two flanges were used to fix the filter/mask samples in place. A digital pressure transducer with a pressure range of 0–1000 Pa was used to measure differential pressure across the samples, and two pressure probes were inserted at a distance of 0*.*5 inch on upstream and downstream of the sample. A handheld vane anemometer was mounted at downstream of the flow to measure the average flow rate. National Instruments’ Data Acquisition (DAQ) system was used to record data from the pressure transducer to the computer. The length of the pipe was 2 m to achieve fully developed flow for given Reynolds number based on velocity cases, keeping in mind the experimental constraints. Pressure resistance versus velocity trends are analyzed from data collected, to determine the breathability of the filters and masks.

#### Virus filtration test

For virus filtration test, bacteriophage MS2 (ATCC 15597-B1) and Escherichia coli C-3000 (ATCC 15597) are used as the test virus and virus host bacteria, respectively. Morphologically, MS2 is a non-enveloped, icosahedral, positive-stranded RNA virus^40^. MS2’s small size, and lack of outer lipid envelope makes it resistant to chemical and environmental resistances. It is used as a surrogate virus for human coronavirus in the current study. The filter/mask samples tested on the virus filtration test bed were a surgical mask and the Hy–Cu 5 layer filter. The idea was to test the novel filter against commercially and widely available surgical masks with comparable pressure drops as observed in pressure resistance test. Also, a single non-woven layer from each sample, namely a surgical mask and the Hy–Cu filter, was separately tested on this test bed with same experimental conditions to check the concentration of virus retained on the non-woven layer after the run.

**Figure 3:**
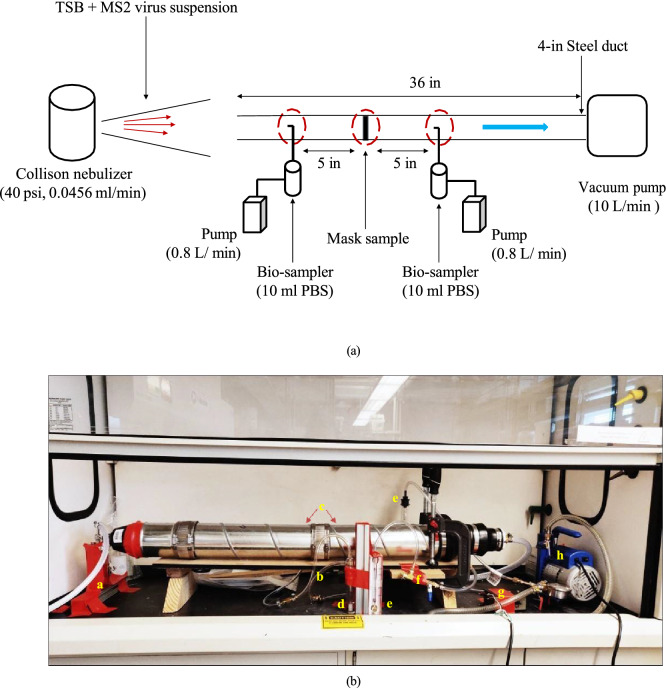
(**a**) Schematic of virus filtration test set up, (**b**) Experimental set up for virus filtration test in fume hood, at BSL-2 laboratory (a: Collison nebulizer; b: test sample; c: bio-sampler probes; d: bio-sampler; e: rotameter; f: valve; g: bio-sampler pump; h: vacuum pump).

The test bed design given in Fig. 3 consists of a Collison nebulizer (CH Technologies Inc.) that sprays aerosolized MS2 bacteriophage suspended in tryptic soy broth (TSB). The nebulizer is set to produce aerosols varying from 0*.*05 to 20 µm at 40 psi pressure and 0*.*0456 ml/min flow rate. The nebulizer was running for 15 min for every test case for sample collection. Virus infused aerosol, from the nebulizer entered a 4-inch duct, 36-inches long, with diverging inlet to prevent back flow of the micro-droplets. The sample filter/mask was fitted at the center of the duct. At a 5-inch distance, upstream and downstream of the filter, two SKC fritted bio-samplers were placed for collection of virus sample. The bio-samplers contained 10 ml of phosphate buffer solution (PBS) for virus aerosol collection and the bio-sampler pumps were run at 0*.*8 L/min. The fritted samplers were run one at a time to prevent interference in sampling and to avoid non-uniform particle spread through duct cross-section, due to the wake region of the probe heads of the bio-samplers, inside the duct. This was obtained through ANSYS Fluent simulations shown in the Supplementary Fig. S2. A vacuum pump was placed at the downstream of the system and was running at 10 times the flow rate of the bio-samplers to drive the flow through the system. Rotameters and ball valves were used to measure and control flow rate of both the vacuum pump and the bio-sampler pump.

For plaque assay analysis of the collected virus sample, soft tryptic soy agar (TSA) was mixed in DI water, and the solution was poured into Petri dishes and allowed to sit until solidified. The collected virus sample and host bacteria were mixed and poured into the Petri dish and incubated overnight at 36 °C. The number of plaques that appeared on the plate were counted and the viral log reduction for the two sample cases, was calculated using the equation,

1 $$LRV = \log_{10} \left( {\frac{{PFU_{downstream} }}{{PFU_{upstream} }}} \right),$$

where LRV is the virus log-reduction value, the numerator is the number of plaque forming units (PFU) on the nutrient agar plate after incubation from the virus sample collected downstream of the filter, and the denominator is the number of plaque forming units on the nutrient agar plate after incubation from the virus sample collected upstream of the filter. The single non-woven layer from a surgical mask and the Hy–Cu filter was tested on the virus filtration bed with consistent experimental conditions to check the concentration of virus retained on the non-woven layer after 15 min of exposure to the aerosolized virus droplets. This was to determine how the overall filtration efficiency was affected by filtration property of non-woven fibers, for better comparison between the two test cases.

#### Virus inactivation test

The virus filtration test bed as shown in Fig. 3 was used to test the performance of the middle layer, Diamond Like Carbon (DLC) coated copper mesh, for virus inactivation. For this, the Hy–Cu 5 layer filter with and without the DLC layer was tested for consistent experimental conditions as discussed in “Virus Filtration test” Section. Two test pieces of size 4 cm × 2cm was cut from the main sample (for Hy–Cu filter with and without DLC) after the 15 min run. One test piece was prepared for the plaque assay test at time t = 0 h, and the other test piece was kept in safe laboratory environment for 2 h, after which it was tested for time t = 2 h. This study was conducted to quantify the role of DLC layer in causing virus inactivation in the filter system.

## Results and discussion

The novel Hy–Cu filter has been tested for breathability, virus filtration and inactivation efficiencies. The plot in the Supplementary Fig. S3 shows the pressure drop trends for the KN95 mask, surgical mask, Hy–Cu 3 layer filter and Hy–Cu 5 layer filter for various velocities. It is shown from the plots that pressure drop across the KN95 mask is much higher as compared to all other sample cases indicating that it is more difficult to breathe through KN95 masks. The Hy–Cu filters offer a resistance ratio (ratio of pressure resistance of the Hy–Cu 5 layer filter to the surgical mask) ≤ 0.9 for flow velocities less than 10 ms^−1^, which is typical for most physical activities^41,42^. At higher flow velocities, both Hy–Cu filter configurations have shown comparable pressure resistance as the surgical mask. It can be noted that the KN95 mask has higher pressure drop compared to both surgical mask and Hy–Cu filters, at higher velocities. These results indicate that it is easier to breathe through the novel Hy–Cu filter as compared to the commercial medical masks. To support the experimental observations with numerical data, the Hy–Cu filter is modeled as porous media and numerical simulations are performed using ANSYS Fluent to extend the pressure drop over wide range of velocities. The detail of the numerical work is given in Supplementary Note 1 of Supplementary Information. The results for numerical simulations as compared to experiments is represented in the plots in Supplementary Fig. S4, for each sample case. The numerical results show good agreement with experimental data with an error of ≤ 30% for lower Reynolds number and improves with increasing Reynolds number.

For the virus filtration test, the virus log reduction values for the Hy–Cu 5 layers and surgical mask are observed to be − 1.2 and − 1.9 respectively. This means that Hy–Cu 5 layers can filter 90% of MS2 bacteriophage virus in 15 min while the surgical mask can filter 95% of the nano-scale virus. To understand the difference in the filtration efficiencies, the non-woven layers from each sample are compared. The non-woven layer is a key factor in the straining, interception, inertial impaction, and diffusion processes that make the particles cling to the fibers. We obtain that the non-woven layer used in the Hy–Cu filter has 2*.*3% virus concentration retention as compared to the non-woven in the disposable surgical mask that shows 1*.*6% of viral concentration after the 15 min of experiment run. This means that the non-woven layers in the Hy–Cu filter are efficiently holding up the virus particles from passing through the filter. The overall lower efficiency of the Hy–Cu filter can be attributed to few points as discussed here. The layers of the Hy–Cu filter are combined together for the experimental tests in laboratory conditions without proper infusion between the layers, whereas for the disposable masks available in market, commercial melt-blown processes are used for creating the mask system. The infusion between the layers of commercially produced masks is better as compared to the Hy–Cu filter system prepared in the laboratory. All three layers of the disposable surgical masks have polypropylene in combination with non-woven material making all the layers better in the hydrophobic property as compared to the novel filter. This can also be a reason for its lower overall efficiency against virus as compared to the medical mask. Also, there is no electrostatic charge applied to the Hy–Cu filter, which is present in disposable surgical masks, that helps in higher overall viral retention increasing the virus filtration capacity of the surgical masks.

For virus inactivation test, the Hy–Cu filter with DLC shows orders of magnitude difference in virus concentration reduction as compared to no DLC condition. The Hy–Cu filter with DLC is observed to have 99% viral inactivation efficiency as compared to no DLC case that shows << 90% of viral inactivation over a period of 2 h. This indicates that the DLC layer has a promising action against MS2 bacteriophage virus. Also, the higher virus retention capacity of non-woven layers present in the Hy–Cu filter is favorable since this subjects the viruses in close proximity to the DLC copper layer leading to eventual inactivation of the virus particles. The overall performance of novel Hy-Cu filter is summarized in Table 1.

**Table 1:** Performance of novel Hy–Cu filter.

Tests	Observations
Pressure resistance	For flow velocities less than 10 m/s, Hy–Cu filter has lower pressure drop (higher breathability) as compared to KN95 and surgical masks
	For higher flow velocities, Hy–Cu filter has comparable performance to surgical mask and better performance than KN95 mask
Virus filtration	Hy–Cu 5 layer filter has 90% viral filtration efficiency
	Surgical mask has 95% viral filtration efficiency
Viral inactivation	In presence of DLC layer, Hy–Cu filter shows 99% virus inactivation ver 2 h
	In absence of DLC layer, Hy–Cu filter shows less than 90% virus inactivation over 2 h
Fluid resistance	Comparable performance to surgical mask and better performance than KN95 mask (discussed in detail in Supplementary Note 2)

The results indicate that the novel Hy–Cu filter is sustainable for use as compared to single-use disposable masks. The better pressure resistance efficiency for Hy–Cu filter as compared to other medical grade masks ensures that it can be used longer, since lower pressure drop across the filter media prevents the early formation of cake layer of dust and other unwanted particulate in air, that eventually clog any filter/mask^44^. Hence the production and disposal of PPE waste materials and challenges associated with it can be controlled. Hewawaduge et al.^45^ shows that CuS (copper sulfide) incorporated three-layer mask has high inactivation efficacy against SARS-CoV-2 virus. Another study^46^ shows that copper coated KF94 masks show promise as antiviral protective face-piece for SARS-CoV-2 inactivation. Our study is the first to show the use of DLC coating on a fine copper mesh for virus inactivation and the use of DLC coating in a mask system. The property of inactivation of virus due to the DLC copper layer makes the Hy–Cu filter highly reusable and safe to use unlike the commercially available disposable masks. This reduces the needs for frequent mask disposal, and in turn bringing down the quantity of PPE, plastic and other related wastes that are being produced every day.

## Conclusion

**Figure 4:**
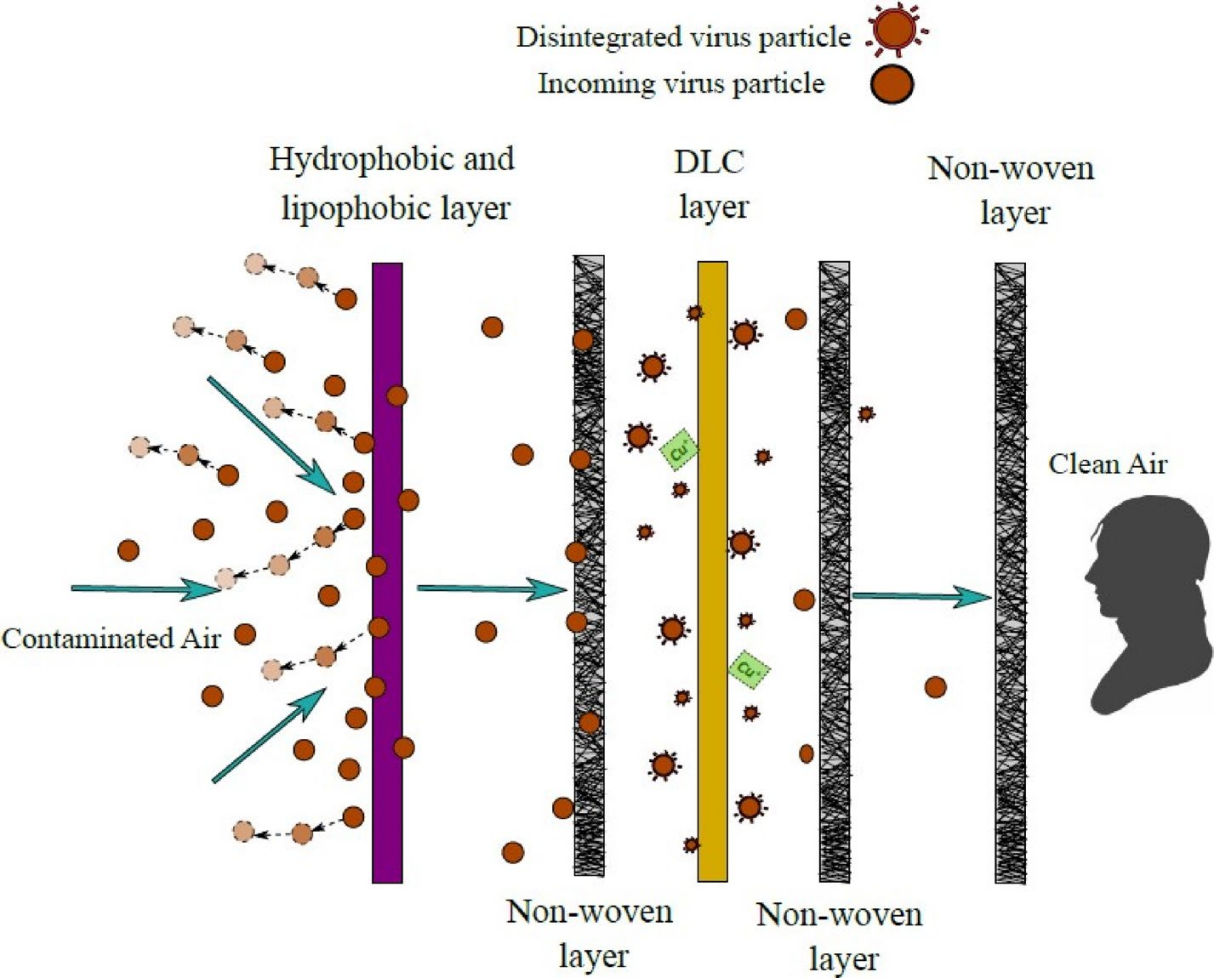
Physical and bio-chemical action of layers of Hy–Cu filter^43^.

Our study is the first to propose a novel filter design. Figure 4 shows the physical and bio-chemical action of each layer of the novel Hy–Cu filter. The novelty of the design lies in the use of a layer coated with in-house hydrophobic and lipophobic coating to repel the coronavirus with outer lipidic envelope and the pH sensitive diamond-like carbon coating on copper mesh, that can lead to virus inactivation process, due to the anti-viral properties. These layers along with non-woven layer can be arranged in different configurations. In this study, we have experimentally and computationally tested the 3 layer and 5 layer configuration of Hy–Cu filter for pressure resistance, and compared it to the medical grade masks. The Hy–Cu 3 layer is highly breathable, followed by Hy–Cu 5 layer, followed by surgical and KN95 mask, over wide range of velocities that involve almost all human activities. The experimental data is used to extract viscous and inertial resistance values that help in characterizing the filter fibers modelled as porous media for numerical modeling of such flows in ANSYS Fluent. These values can be used to create more realistic cases of simulations for masks by designing geometries with the an-isotropic fiber arrangement as opposed to our study where the porous zone is designed as a simple cylinder. Our simulations are in good agreement with experimental data. In terms of virus filtration efficiency, the surgical mask and Hy–Cu 5 layer filter is subjected to aerosol droplets carrying MS2 virus, in well controlled experiments. The novel filter shows 90% efficiency, and the disposable surgical mask shows 95% efficiency. The Hy–Cu filter performs very well in terms of virus inactivation over a period of 2 h, in the presence of DLC layer, observing 99% efficiency. Future work can be done to characterize the kinetic decay of virus over time for DLC layer. This may help in reducing the re-usability time to lower than 2 h. Our study in this work is limited to test the filter when a susceptible person wearing the filter is exposed to infected droplets carrying the virus through the outermost layer. The future work can be extended to study the case where an infected person is wearing the filter and flow of virus droplets is from inside layer to outside. The novel Hy–Cu filter is an environmentally judicious and efficient substitute to the disposable masks currently used. It has very high performance in terms of inactivation, breathability and longer usability as well as virus filtration. The flexible design characteristics and reusable property, makes this a unique contribution to combat the current and future pandemic phases.

## Supplementary Information

Supplementary Information.
