# Supplementary material for: Novel sustainable filter for virus filtration and inactivation
Source: Sci Rep. 2022 Jun 1;12:9109. doi: 10.1038/s41598-022-13316-9 (PMC9156824; doi:10.1038/s41598-022-13316-9)
Supplement: Supplementary file 1 — Supplementary Information. [file 41598_2022_13316_MOESM1_ESM.docx]

# Supplementary Information

## Supplementary Figure 1


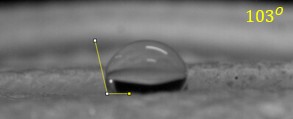

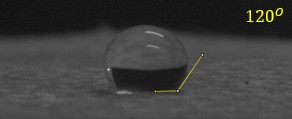

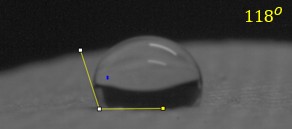


1. Surgical mask (b) N95 mask (c) Hy-Cu filter

**Figure S1.** Contact angle of artificial saliva droplet on external layer of sample masks and filter

## Supplementary Figure 2


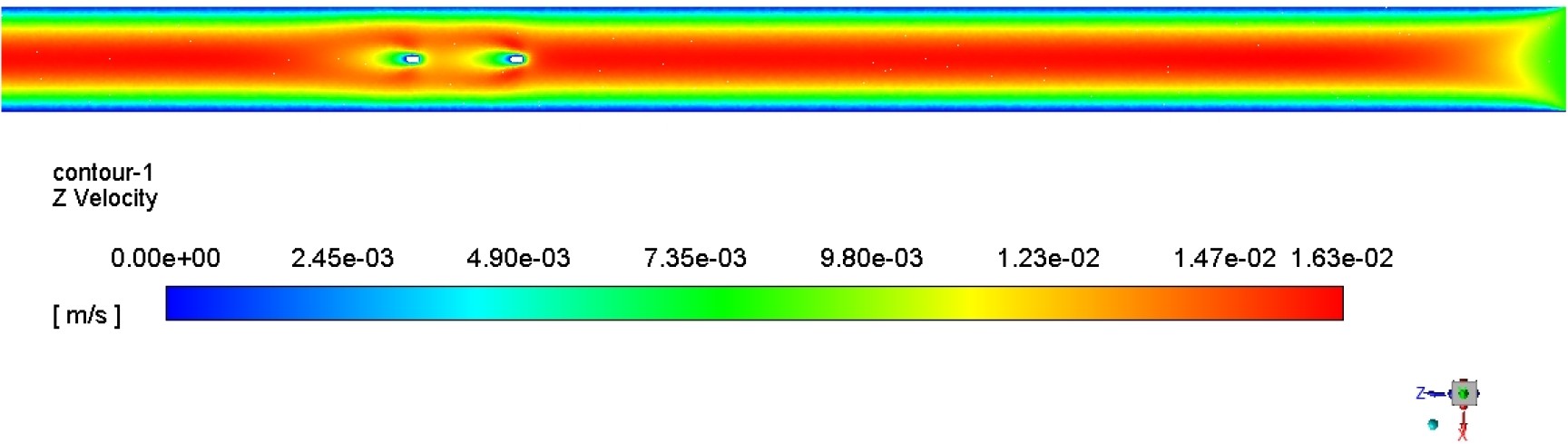


**Figure S2.** ANSYS Fluent simulations to show the non-uniform particle distribution in the presence of pressure probes

## Supplementary Figure 3


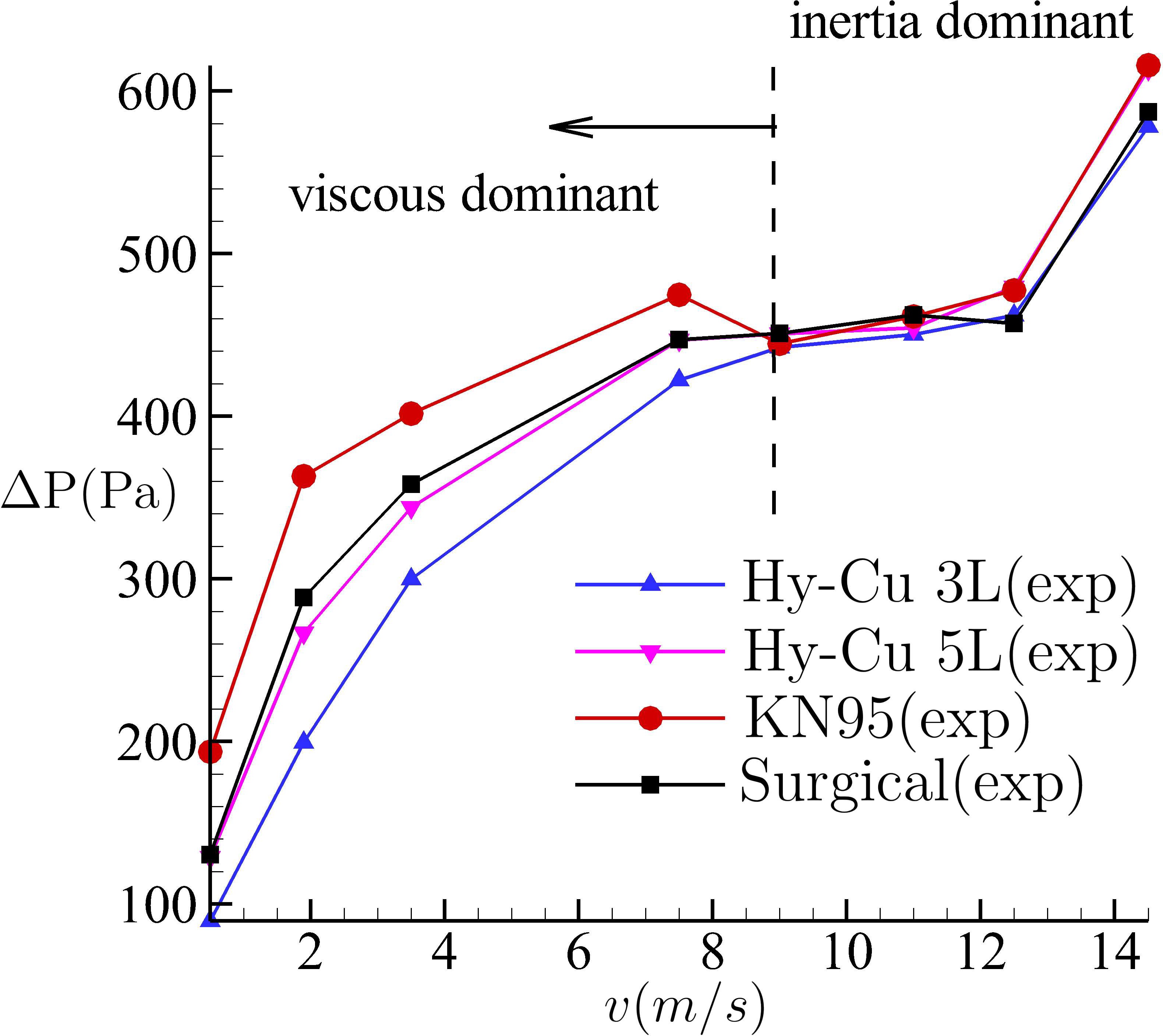


**Figure S3.** Comparison of pressure resistance vs velocity for sample cases

## Supplementary Figure 4

The pressure drop on y-axis is normalized with dynamic pressure head (0*.*5*ρv*^2^) while the velocity is normalized as Reynolds number, where *Re_D_* and *Re_t_* represent the Reynolds number with respect to the pipe diameter (25*.*4*mm*) and porous media thickness (2*mm*) respectively.


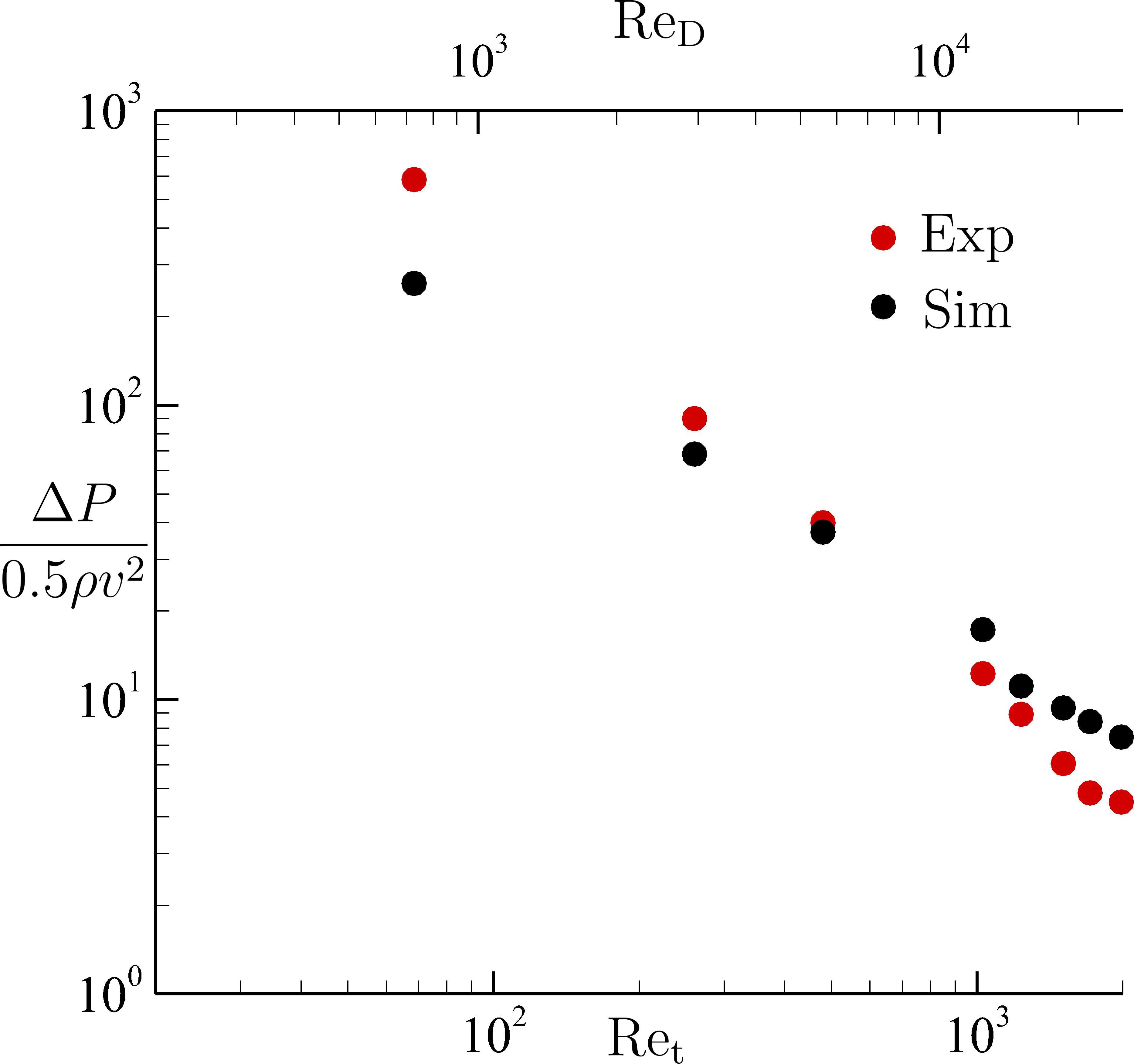

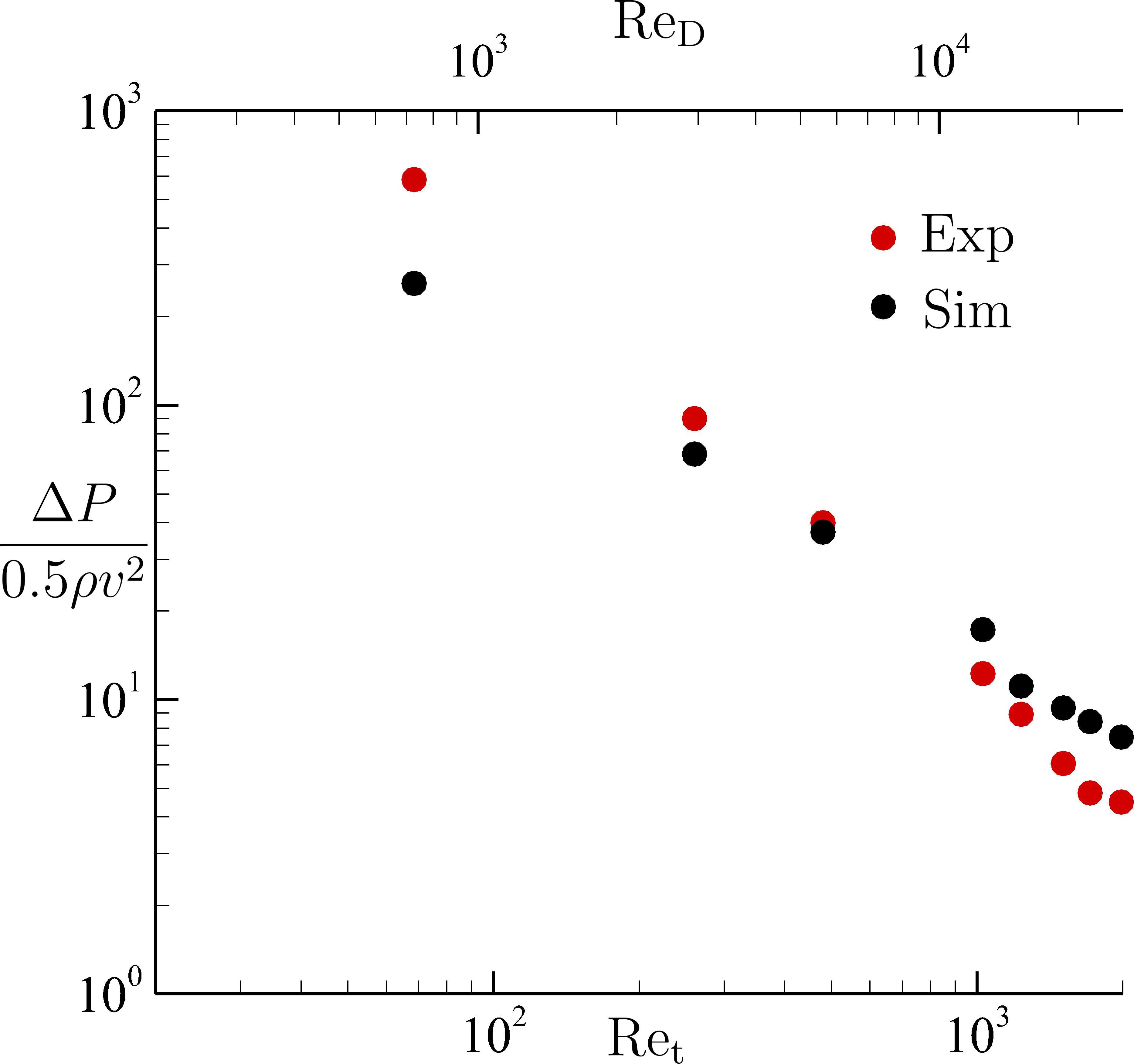


- 1. Surgical mask (b) N95 mask


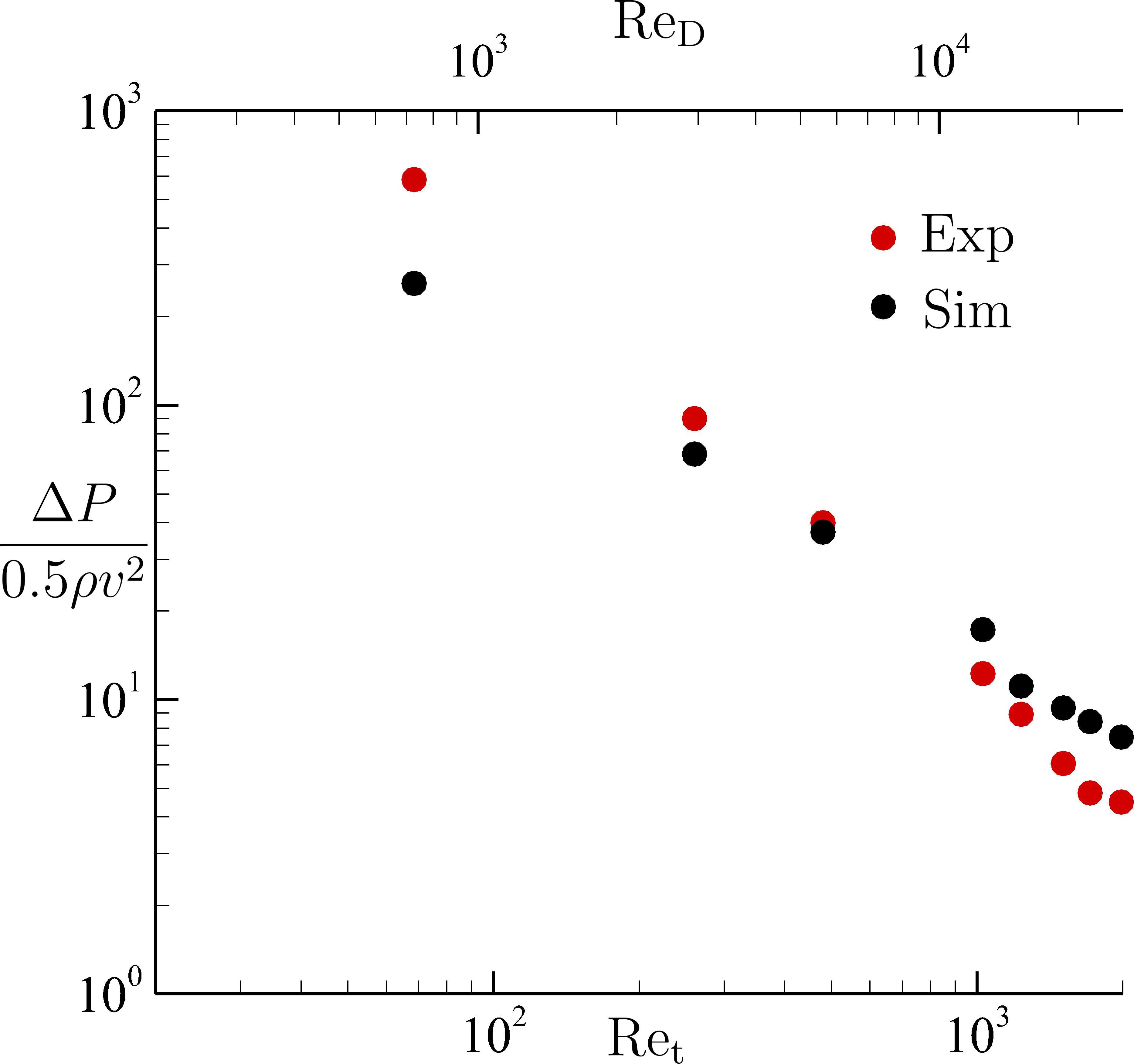

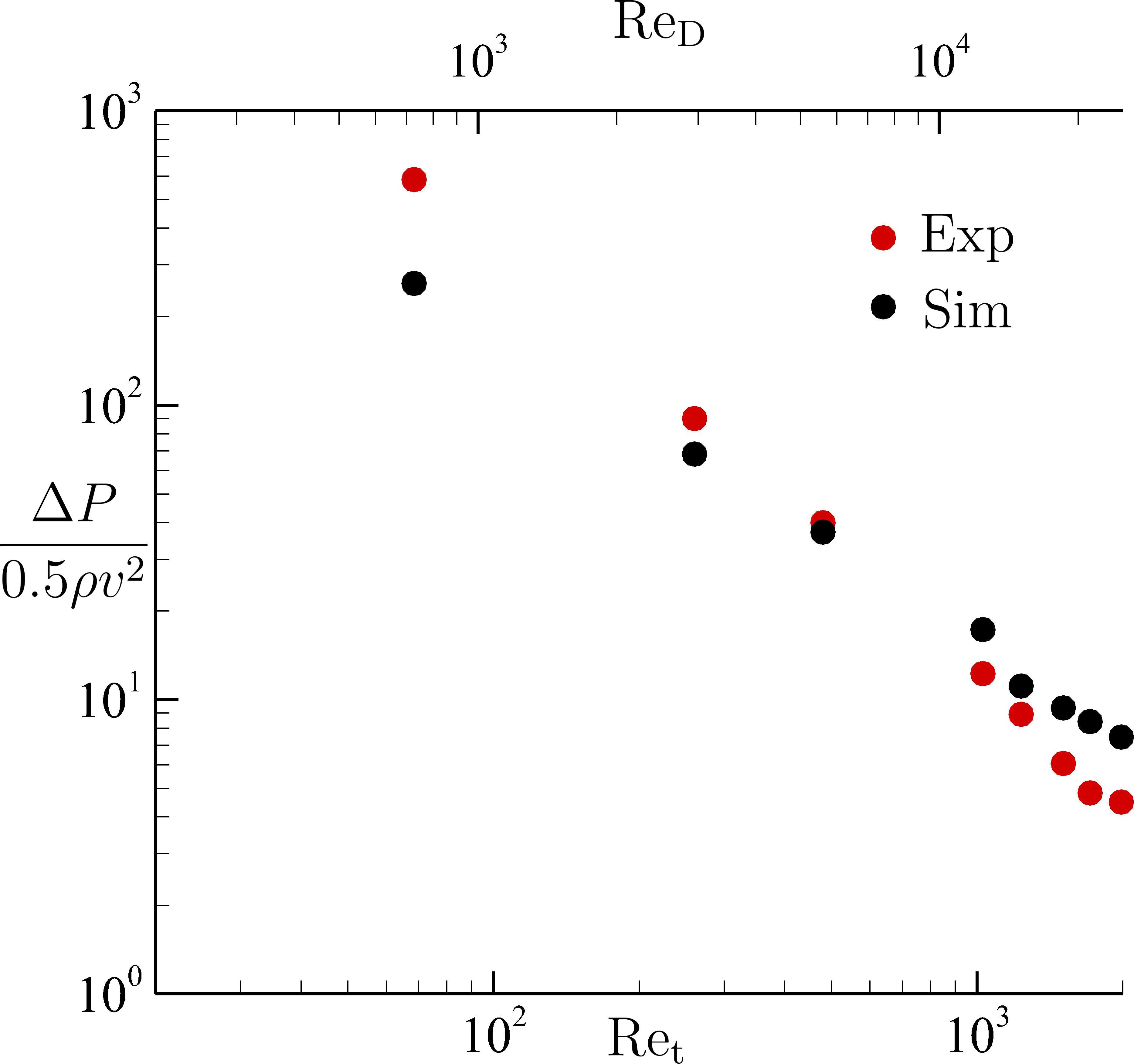


(c) Hy-Cu filter (d) Surgical mask

**Figure S4.** Comparison of normalized pressure resistance vs Reynolds number for sample cases

## Supplementary Note 1 (Computational Study Method)

The fibrous filter/ mask is numerically modeled as porous zone, in ANSYS Fluent. Porosity of the fibrous filter is one of the most crucial parameters strongly affecting both collection efficiency and pressure drop for a mask that behaves like porous media (ANSYS user guide: Porous Media Conditions). Energy is lost in terms of viscous and inertial resistance when a flow passes through porous media. In such flows, for a given energy input (measured as pressure drop), it is useful to be able to predict the flow rate or to be able to predict pressure drop from a given flow rate. Dullien suggested a general physical model that total pressure drop or flow resistance is a sum of viscous losses and inertial losses[^1^](#_bookmark6), like a truncated power series for pressure drop as a function of velocity,

*−*∇*P* = *C*_1_*U* +*C*_2_*U* ^2^*,* (1)

where, *C*_1_ is the viscous coefficient and *C*_2_ is the inertial coefficient. In modeling of porous media, a momentum source term

(S) is added to the Navier-Stokes equation for a fluid flow as equation,

*∂* (*ρU_i_*) + *∂* (*ρU_i_U_j_*) = *− ∂ p* + *∂*  *µ* *∂Ui* + *∂Uj* *−* 2 *µ*( *∂Ui* )*I* + *ρg* + *S.* (2)

*∂t*

*∂x_j_*

*∂x_i_*

*∂x_j_*

*∂x_j_*

*∂x_i_*

3

*∂x_j_*

The source term is composed of viscous loss term and inertial loss term. It is non zero in the porous zone and zero outside the porous zone and written as equation,

*S* = *−*(*C*_1_*U* +*C*_2_ 1 *ρUU*

2

1

) *t .*

(3)

This helps in modeling of the porous media and the additional energy losses due to its presence in the flow. There are dominant viscous losses at lower Reynolds number while dominant inertial losses at higher Reynolds number. For laminar flows, Darcy’s law is a classical equation that describes the flow of a fluid through a porous medium,[^2^](#_bookmark7)

∆*P µU*

*L* = *α .* (4)

This shows that at lower Reynolds number, where the viscous losses are dominant, the pressure drop varies linearly with velocity. For lower flow velocities, the constant *C*_2_ can be considered zero. At high flow velocities, the constant *C*_2_ gives the loss coefficient per unit length in direction of flow, and hence the pressure drop is written as a function of dynamic head. The porous coefficients *C*_1_ and *C*_2_ can be determined from the experimental data that is obtained in the form of pressure drop against velocity in Figure [S3](#_bookmark0). The porous coefficient values are tabulated in Table [S1](#_bookmark1).

## Supplementary Table 1

**Table S1.** Viscous (*C*_1_) and Inertial (*C*_2_) resistance values from experimental data


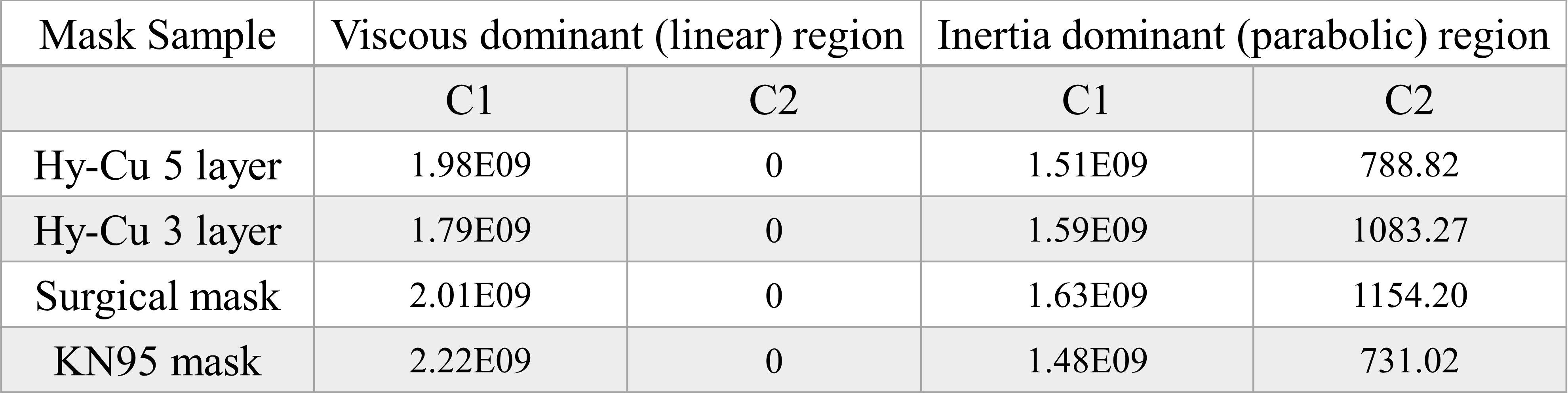


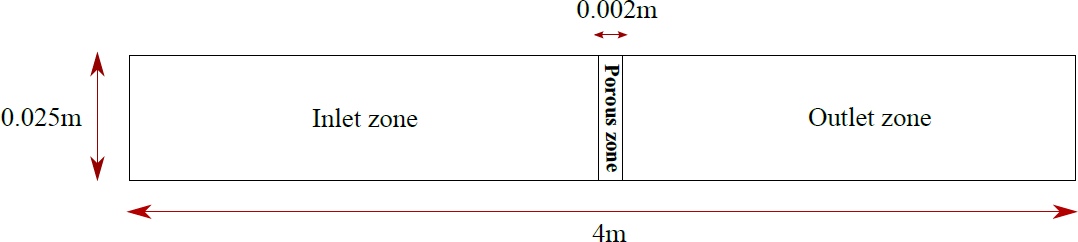


**Figure S5.** Computational set up for pressure resistance test in ANSYS Fluent

The numerical modelling in current study involves a simple pipe flow, with inlet zone, porous zone and outlet zone as shown in Figure [S5](#_bookmark2). The viscous model used in FLUENT is standard k-*ε* with standard wall functions. The fluid material is Air. For boundary conditions, a uniform velocity inlet, pressure outlet and no-slip wall are considered. In the cell zone conditions, the porous zone is characterized based on viscous and inertial resistance values obtained from experimental data. Absolute pressure values are obtained as area weighted average right before and after the porous zone of thickness 2mm, to calculate the pressure

drop. For grid independent study, pressure drop is chosen as the parameter and four meshes of different grid sizes (number of elements) namely, M1 (50k), M2 (100k), M3 (250k), and M4 (400k) are compared. Pressure drop value changes 0*.*0031% (almost negligible) between M3 and M4 and so we chose M3 for accuracy and efficient computational time. Pressure drop results from the ANSYS numerical modeling are compared with the experiment.

## Supplementary Note 2 (Qualitative Efficiency)

A comparative qualitative study is performed to determine the fluid resistance performance of the medical masks and novel Hy-Cu filter, considered a standard test for determining mask effectiveness[^3^](#_bookmark8). As shown in the schematic in Figure [S6](#_bookmark4), the samples are exposed to a Collison nebulizer spraying colored artificial saliva droplets ranging from 0.005 to 20 microns for 10 minutes at a maximum pressure of 100 psi. The distance between the nozzle and the sample mask is 1 inch and the vacuum pump operates at 6L/min to keep human inhalation effects in consideration.


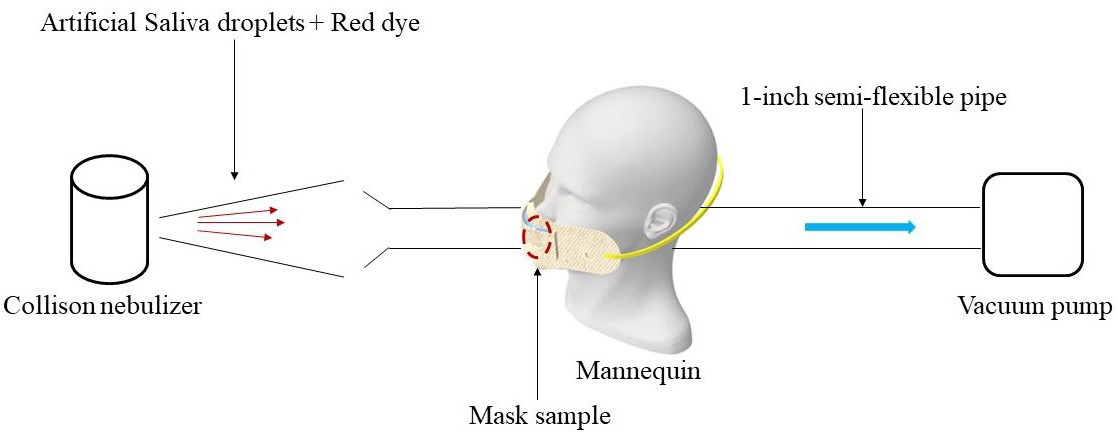


**Figure S6.** Schematic diagram for fluid resistance study

As seen in Figure [S7](#_bookmark5), no spotting is observed through the surgical disposable mask, slight spotting is observed through the KN95 mask and no spotting is observed through the novel Hy-Cu filter. This shows that the novel filter is resistant to fluid sprayed at high pressure and is safe and effective specially in healthcare settings where patients are often coughing or sneezing.


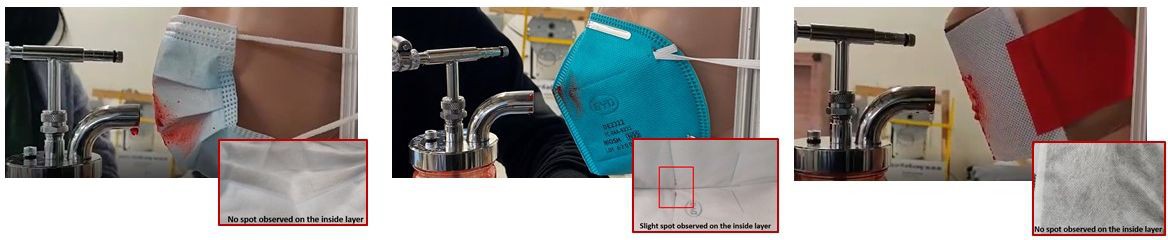


**Figure S7.** Comparison of surgical mask, KN95 mask and Hy-Cu filter for qualitative efficiency

# References

1. Dullien, F. Single phase flow through porous media and pore structure. *The Chem. Eng. J.* **10**, 1–34 (1975).
2. Whitaker, S. Flow in porous media i: A theoretical derivation of darcy’s law. *Transp. porous media* **1**, 3–25 (1986).
3. Ju, J. T., Boisvert, L. & Zuo, Y. Y. Face masks against covid-19: Standards, efficacy, testing and decontamination methods.

*Adv. Colloid Interface Sci.* 102435 (2021).
